# Cohort Profile: Indian Study of Healthy Ageing (ISHA-Barshi)

**DOI:** 10.1093/ije/dyae079

**Published:** 2024-06-14

**Authors:** Sharayu Sitaram Mhatre, Fiona Bragg, Nandkumar Panse, Parminder Kaur Judge, Ankita Manjrekar, Julie Ann Burrett, Suchita Patil, George Davey Smith, Lekha Kotkar, Caroline L Relton, Pravin Doibale, Bipin Gadhave, Pankaj Chaturvedi, Paul Sherliker, Prabhat Jha, Sarah Lewington, Rajesh Dikshit

**Affiliations:** Division for Molecular Epidemiology and Population Genomics, Centre for Cancer Epidemiology, Tata Memorial Centre, Kharghar, Navi Mumbai, India; Homi Bhabha National Institute (HBNI), Mumbai, India; MRC Population Health Research Unit, Nuffield Department of Population Health, University of Oxford, Oxford, UK; Clinical Trial Service Unit and Epidemiological Studies Unit, Nuffield Department of Population Health, University of Oxford, Oxford, UK; Health Data Research UK Oxford, University of Oxford, Oxford, UK; Division for Molecular Epidemiology and Population Genomics, Centre for Cancer Epidemiology, Tata Memorial Centre, Kharghar, Navi Mumbai, India; Clinical Trial Service Unit and Epidemiological Studies Unit, Nuffield Department of Population Health, University of Oxford, Oxford, UK; Division for Molecular Epidemiology and Population Genomics, Centre for Cancer Epidemiology, Tata Memorial Centre, Kharghar, Navi Mumbai, India; Clinical Trial Service Unit and Epidemiological Studies Unit, Nuffield Department of Population Health, University of Oxford, Oxford, UK; Division for Molecular Epidemiology and Population Genomics, Centre for Cancer Epidemiology, Tata Memorial Centre, Kharghar, Navi Mumbai, India; MRC Integrative Epidemiology Unit, University of Bristol, Bristol, UK; Population Health Sciences, Bristol Medical School, University of Bristol, Bristol, UK; Division for Molecular Epidemiology and Population Genomics, Centre for Cancer Epidemiology, Tata Memorial Centre, Kharghar, Navi Mumbai, India; MRC Integrative Epidemiology Unit, University of Bristol, Bristol, UK; Population Health Sciences, Bristol Medical School, University of Bristol, Bristol, UK; London School of Hygiene & Tropical Medicine, London, UK; Division for Molecular Epidemiology and Population Genomics, Centre for Cancer Epidemiology, Tata Memorial Centre, Kharghar, Navi Mumbai, India; Division for Molecular Epidemiology and Population Genomics, Centre for Cancer Epidemiology, Tata Memorial Centre, Kharghar, Navi Mumbai, India; Division for Molecular Epidemiology and Population Genomics, Centre for Cancer Epidemiology, Tata Memorial Centre, Kharghar, Navi Mumbai, India; Homi Bhabha National Institute (HBNI), Mumbai, India; MRC Population Health Research Unit, Nuffield Department of Population Health, University of Oxford, Oxford, UK; Clinical Trial Service Unit and Epidemiological Studies Unit, Nuffield Department of Population Health, University of Oxford, Oxford, UK; Centre for Global Health Research (CGHR), St Michael's Hospital and Dalla Lana School of Public Health, University of Toronto, Toronto, ON, Canada; MRC Population Health Research Unit, Nuffield Department of Population Health, University of Oxford, Oxford, UK; Clinical Trial Service Unit and Epidemiological Studies Unit, Nuffield Department of Population Health, University of Oxford, Oxford, UK; Health Data Research UK Oxford, University of Oxford, Oxford, UK; Division for Molecular Epidemiology and Population Genomics, Centre for Cancer Epidemiology, Tata Memorial Centre, Kharghar, Navi Mumbai, India; Homi Bhabha National Institute (HBNI), Mumbai, India

Key Features

- The Indian Study of Healthy Ageing (ISHA) examines the burden, causes and consequences of chronic diseases in an Indian adult population of ∼220 000. Once completed, ISHA will be the largest blood-based prospective study in South Asia. Expansion to sites in Varanasi, Guwahati, Sangrur and Mullanpur will further strengthen the study.
- Recruitment began in 2015 in Barshi, Maharashtra state, India. Enumeration, including basic baseline data collection, has been completed for all participants. Further detailed data collection, through questionnaires, physical measurements and blood sampling, is complete for ∼39 000 participants and ongoing. Active follow-up and linkage to health-related datasets (for death, cancer and hospitalization) provide data on fatal and non-fatal disease events.
- At baseline among the ∼39 000 participants, 53% were women, and mean (standard deviation: SD) age was 46 (11). Tobacco smoking and alcohol drinking are relatively uncommon, but tobacco chewing is prevalent (67% of men and 14% of women ever chewed tobacco). The population is relatively lean, with a mean (SD) body mass index of 23.2 (4.1) kg/m^2^. Self-reported, previously diagnosed chronic diseases were uncommon (hypertension: 6%; diabetes: 3%; cardiovascular disease: 1%; cancer: <1%). Depressive symptoms were common (at least one reported by 58%).
- Data-sharing regulations are in place; specific proposals for future collaboration are welcomed.

## Why was the cohort set up?

The Indian Study of Healthy Ageing (ISHA) is a blood-based prospective cohort study of approximately 220 000 individuals aged 30–69, recruited between 2015 and 2020 from towns and villages around Barshi, Solapur district, Maharashtra state, India (Supplementary Figure S1, available as Supplementary data at *IJE* online). The study was established with the aim of testing hypotheses relating to lifestyle, diet, obesity and related factors, and of identifying potential genetic determinants of cancer and other chronic diseases in a general population with low baseline risk. Table 1 shows 2019 age-standardized mortality rates for middle-aged men and women in Barshi, India, the USA and the UK. All-cause mortality was notably higher in Barshi than in the USA and UK, but was moderately lower than India’s national mortality rates, particularly among women. Vascular disease accounted for about one-third of premature adult mortality in Barshi, and ischaemic heart disease accounted for a substantially higher proportion in Barshi than in the UK and USA. There were also notably higher respiratory disease mortality rates in Barshi, although rates were lower than in India overall among both men and women. Previous studies in India have reported similar patterns of vascular disease,^1^ but much remains unknown about factors underlying these recent trends in vascular mortality. ISHA would be expected to contribute significantly to advancing our understanding of environmental and genetic risk factors for vascular and non-vascular mortality in the Indian population. Major transitions in lifestyle behaviours have been seen in India and these continue, particularly in rural areas; a further aim of ISHA is to understand the relevance of these for common chronic diseases. Collection and long-term storage of blood samples will allow investigation of genetic susceptibility to cancer and other chronic diseases and of the relevance of other blood-based risk factors, which might conceivably differ from those in European ancestry populations.

**Table 1.** Age-standardized mortality rates^a^ at ages 30 to 69 for Indian Study of Healthy Ageing study areas in 2019 and, for comparison, India, USA and UK^b^

	**Annual deaths per 100** **000**							
	Men				Women			
Cause of death	Barshi	India	USA	UK	Barshi	India	USA	UK
IHD	258	245	128	89	171	134	48	26
Stroke	50	97	26	19	33	79	19	14
Cancer	122	137	218	203	116	133	168	172
Diabetes	24	35	23	5	10	31	14	3
Lung disease	59	126	38	29	43	90	31	23
Other	448	458	286	158	271	339	155	93
All causes	962	1097	719	503	644	806	435	331

a Rates are age-standardized by taking the unweighted average of the component 5-year mortality rates (e.g. 30–34, 35–39, … 65–69 for the age range 30–69 years).

b Source: Global Burden of Disease Study 2019.

IHD: Ischaemic heart disease.

ISHA was conceived, developed and is coordinated by the Centre for Cancer Epidemiology (CCE), based at the Tata Memorial Centre, Kharghar, Navi Mumbai, India. Following an initial pilot study (funded by the International Agency for Research on Cancer, Lyon, France and the Centre for Global Health Research, Canada), the main study, including establishment of an automated biobank (for storing 3 million samples), is funded by Tata Memorial Centre.

## Who is in the cohort?

Participants were recruited from 362 villages and three small towns around Barshi (Warshi, Bhum, Paranda). Barshi was chosen given its relatively stable population, with little migration in and out of the region, and previous experience demonstrating the feasibility of long-term follow-up in the area. A Regional Coordinating Centre was established, led by medical and field coordinators (Supplementary Figure S2, available as Supplementary data at *IJE* online). Potentially eligible participants were identified through official residential records, and field coordinators visited their households. To encourage participation, all household members in the target age range (30–69 years) were eligible for enrolment into the study. Of 128 897 eligible households, 119 387 (93%) participated (Supplementary Table S1, available as Supplementary data at *IJE* online). Within participating households, 93% (*n* = 219 888) of eligible individuals were recruited to the study, with age and sex distributions similar to those of the 17 362 household members who were not recruited (Supplementary Table S2, available as Supplementary data at *IJE* online). After an enumeration process, fieldworkers visit participants’ homes to complete a detailed Household Health Survey interview. Since study villages do not have house numbers, unique enumeration numbers are marked on participants’ houses which, along with GPS records, ensure the correct houses are visited for completion of the Household Health Survey. At this time, letters are provided inviting participants to visit temporary Health Check-Up Camps set up inside the villages and towns, where physical measurements and blood samples are taken. As a pre-requisite for participating, all participants are asked to show their unique national identity (ID) cards—Aadhaar cards^2^—to fieldworkers when they visit their homes. Of the 38 442 participants who have completed the Household Health Survey to-date, 30 398 (79%) have attended the Health Check-Up Camp, with no significant differences in sociodemographic and lifestyle characteristics of participants according to attendance.

All participants provided informed consent, which allows access to participants’ medical records and long-term storage of blood for anonymized and unspecified medical research purposes.

## How often have they been followed up?

Periodic resurveys of reasonably representative samples of ∼10 000 participants will be performed every 5–10 years (Supplementary Figure S3, available as Supplementary data at *IJE* online). These will be important for assessing temporal trends, e.g. in lifestyle and sociodemographic factors, given the ongoing rapid development in rural India, as well as providing opportunity for further enhancement of data collection. Moreover, these resurveys will enable assessment of within-person variation in exposures, and correction for resulting ‘regression dilution’ bias.^3^

Vital status of participants is being monitored indefinitely based on manual linkage to death registries and through active follow-up (Supplementary Figure S4, available as Supplementary data at *IJE* online). Verbal autopsies are conducted by study staff to determine the most likely cause of death.^4^^,^^5^ Further manual linkage to cancer registries,^6^^,^^7^ primary health care and hospital registers (employed in these established cancer registries), and the Rajeev Gandhi Health Insurance Scheme,^8^ in addition to active follow-up, provide data on disease incidence (Supplementary Figure S5, available as Supplementary data at *IJE* online). Additional active follow-up is undertaken approximately every 3–5 years through fieldworker visits to participants’ households (Supplementary Figure S6, available as Supplementary data at *IJE* online). Surviving participants are asked about new diagnoses of diseases since the baseline interview and about hospitalizations during the same period, and data are again collected on certain risk factors, including lifestyle factors. Where deaths have occurred, details are collected from household members, including through verbal autopsy. Collection of the Aadhaar number (a twelve-digit unique identification number^2^) from all participants presents future opportunity for passive linkage to additional health care and non-health care data for longitudinal follow-up.

Overall during the first 6 years of follow-up, <1% of participants (208 out of 38 442) are lost to active follow-up due to migration. When compared with those under active follow-up, these participants are, on average, younger (mean [SD] 42 [11] vs 46 [11] years) and, consistent with this age difference, are more highly educated (36% vs 26% with 6+ years education) and less frequently reported a history of chronic disease (10% vs 14%).

## What has been measured?

Baseline data collection comprises three distinct stages: (i) enumeration (or study registration); (ii) the Household Health Survey; and (iii) the Health Check-Up Camp. The enumeration visit was performed in participants’ homes. After giving written, informed consent, data were collected on household characteristics, including composition, religion, indicators of indoor air pollution, and details of hospitalizations and deaths among the household (Table 2). Following this enumeration process, the on-going Household Health Survey comprises a face-to-face, interviewer-administered questionnaire completed by each participating household member. Interviews are performed using a laptop-based questionnaire with data entered directly into the form using a data entry system developed specifically for the project by the Centre for Global Health Research, Canada. Table 2 summarizes data collected in the questionnaire, including information on age, sex, indicators of socioeconomic status (including education), lifestyle factors (including tobacco smoking and chewing, alcohol drinking—incorporating wives’ reporting of their husbands’ alcohol intake—diet and physical activity), personal and family medical history, sleep and mood. Blood pressure measurements are also undertaken at this time. Subsequently, physical measurements, including repeat blood pressure measurements, anthropometric measures, bioimpedance assessment, pulmonary function tests and handgrip strength (Table 2), are undertaken at village-based Health Check-Up Camps (Supplementary Figure S7, available as Supplementary data at *IJE* online) using standard protocols. All data collection is undertaken by trained personnel, who undergo a biannual programme of training. Nail clippings are collected for assessment of exposure to pesticides and other chemicals. These are shipped at room temperature to CCE, Kharghar, for long-term storage. A 10-mL non-fasting venous blood sample is collected into an EDTA vacutainer. During the initial phases of sample collection, blood samples were placed in portable insulated cool boxes with ice packs at 4 °C and initially processed at a satellite CCE laboratory located in Nargis Dutt Memorial Cancer Hospital, Barshi, India within 24 h of collection. They were aliquoted into five to seven bar-coded cryovials (including one DNA-containing buffy coat) and transported on dry ice to CCE, Kharghar, for long-term storage at −80°C in an automated biobank. The blood sample transport and processing protocol has subsequently been updated to enable transfer of EDTA tubes using battery-operated portable freezers to CCE, Kharghar, on the day of collection by road transport. Samples are fractionated at CCE, Kharghar, and transferred into the same number of barcoded cryovials for storage in the automated biobank.

**Table 2.** Summary of baseline questionnaire and physical measurement data

**Individual participant questionnaire**	
Demographic data	Family history
Name	Parental age/age at death
Age	Parental medical history
Sex	Number of siblings
Marital status	Siblings’ medical history
Socioeconomic data	Sleeping and mood
Education	Life satisfaction
Occupation	Traumatic events
General health-related behaviour	Sleep situation
Self-rated health status	Self-rated mood status
Disease history (13 common conditions)	Reproductive history for women
Current medication	Age at menarche
History of blood transfusions	Menopause status
History of oral leukoplakia/erythroplakia	History of contraceptive pill use
Pattern of bowel movements	History of pregnancy
Exposure to passive smoking	
Body size in childhood and early adulthood	
Health behaviours	
Tobacco smoking (cigarette, bidi)	
Chewing (betel leaf, tobacco)	
Alcohol drinking	
Food frequency questionnaire	
Physical activity (occupational and leisure time)	
**Household questionnaire**	
Size and composition	
Religion	
Caste	
Receipt of government-sponsored scheme	
House type/room number/cooking fuel	
**Physical measurements**	
Resting blood pressure	
Resting heart rate	
Standing and sitting height	
Weight	
Body-mass index	
Waist circumference	
Hip circumference	
Bio-impedance	
Index and ring finger length	
Body composition*	
Retinal imaging*	
Lung function	
Grip strength	

* 10,000 participant substudy

All collected data are entered directly onto laptops. At the end of each day, the data are transferred in an encrypted format to the central study database at CCE, Kharghar (the central coordinating office for the study), using a secured internet connection, at which point the data are deleted from the data collection laptops (Supplementary Figures S8 and S9, available as Supplementary data at *IJE* online). Personal identifiers of participants are stored in separate tables in the database, with limited access. A web portal dashboard provides daily statistics for monitoring of participant enrolment and data collection. Incremental and full back-up of the central database is undertaken on a daily, weekly and monthly basis on CCE, Kharghar, servers.

Repeat baseline assessments are undertaken among a random subset of ∼8% of participants, typically within 3–4 days of the original assessment. These are carried out by a different fieldworker blinded to the original data and provide an opportunity for fieldworker training and quality control.

In ongoing sub-studies, whole-body dual-energy absorptiometry (DXA) scans (providing data on body composition, including fat mass and its distribution) and digital non-mydriatic retinal imaging are being undertaken among 10 000 participants.

## What has it found?

**Figure 1.**
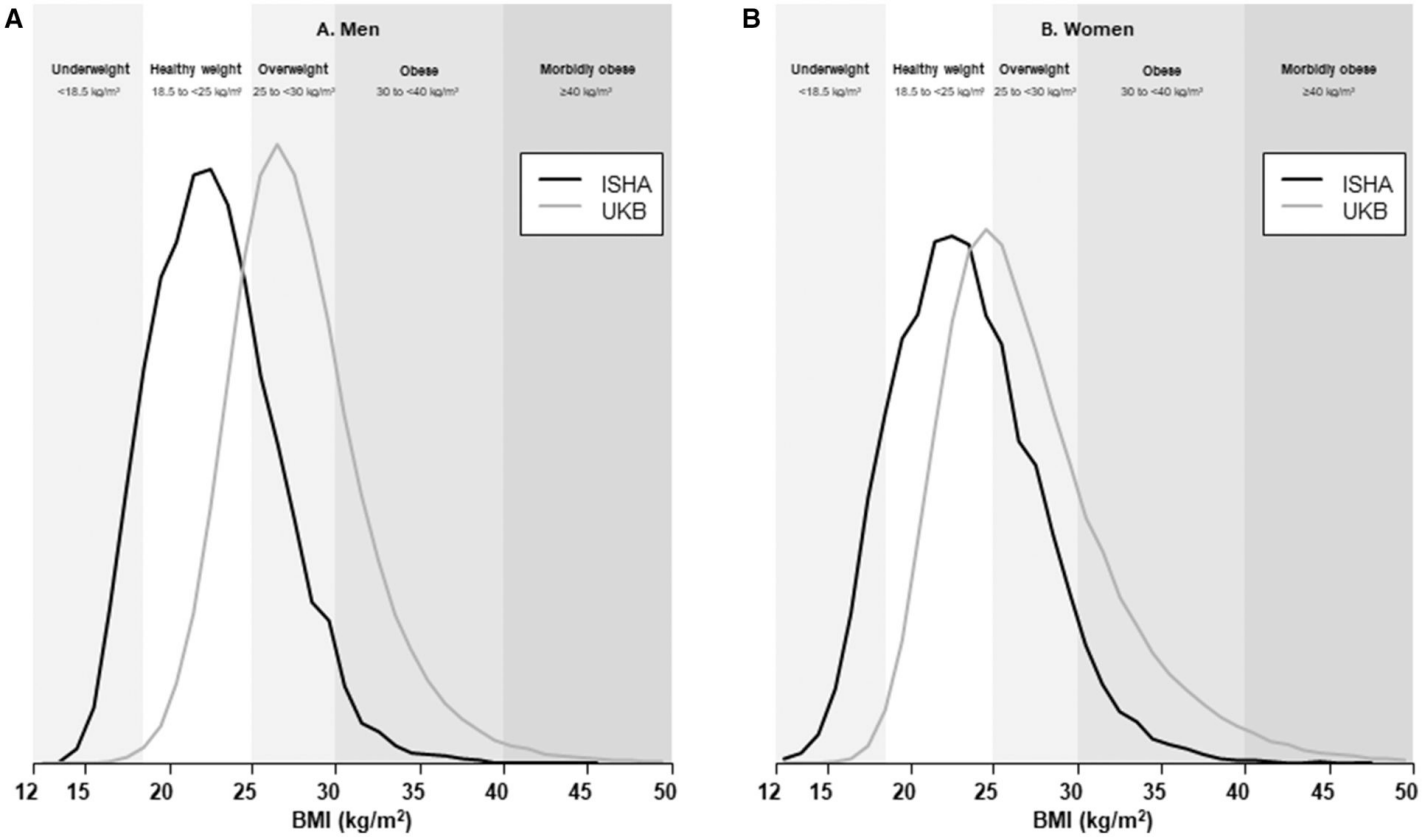
Distributions of body mass index in the Indian Study of Healthy Ageing (*n* = 20 642) and UK Biobank (*n* = 496 845). Age range of ISHA is restricted to 40–69 years in order to match UK Biobank. ISHA, Indian Study of Healthy Ageing; UKB, UK Biobank

Overall, among the 219 888 participants recruited from 119 387 households, the mean (SD) age was 47 (11) years at enumeration with an equal sex distribution (Supplementary Table S2, available as Supplementary data at *IJE* online). Comprehensive baseline data collection has been completed for 38 442 participants (Table 3). Their mean (SD) age at the time of the Household Health Survey was 46 (11) years and 53% were women. Among men, 41% reported completing at least 6 years’ education, in contrast to only 14% of women. Educational attainment was strongly inversely associated with age in both sexes. Two-thirds of men reported that they ever chewed tobacco, with a smaller proportion (8%) reporting ever smoking; both habits were more common among older men. Among women, 14% reported that they ever chewed tobacco, and smoking was rare (<1%). Few women reported drinking alcohol (<1% ever-drinkers), but drinking was more common among men (25%). Mean (SD) body mass index (BMI) was similar among men at 23.0 (3.9) kg/m^2^ and women at 23.3 (4.4) kg/m^2^. The prevalence of underweight (BMI <18.5 kg/m^2^) was also similar in men and women (9% and 10%, respectively), but overweight (25.0 to <30.0 kg/m^2^) and obesity (BMI ≥30.0 kg/m^2^) were slightly more common among women (21% and 6%, respectively) than men (19% and 3%, respectively) (Supplementary Table S3, available as Supplementary data at *IJE* online). These BMI distributions are much lower than those typical of more widely studied Western populations. For example, comparable data from UK Biobank showed <1% of men and women were underweight, 49% of men and 37% of women were overweight and 25% and 24%, respectively, were obese (Figure 1).

**Table 3.** Baseline characteristics of 38 442 study participants, by age and sex

	Men, by age (years)				Women, by age (years)			
	30–49	50–59	60–69	All	30–49	50–59	60–69	All
Number of participants	10 170 (57)	4083 (23)	3710 (21)	17 963 (100)	12 454 (61)	4694 (23)	3331 (16)	20 479 (100)
Age, years	38 (6)	54 (3)	63 (3)	47 (11)	38 (6)	53 (3)	63 (3)	46 (11)
6+ years of education	5287 (52)	1085 (27)	910 (25)	7282 (41)	2566 (21)	198 (4)	100 (3)	2864 (14)
Ever chew tobacco	5922 (58)	3079 (75)	3041 (82)	12 042 (67)	1065 (9)	973 (21)	875 (26)	2913 (14)
Ever smoke	517 (5)	442 (11)	431 (12)	1390 (8)	3 (0.02)	7 (0.1)	5 (0.2)	15 (0.1)
Ever drink alcohol	2268 (22)	1167 (29)	1066 (29)	4501 (25)	11 (0.1)	3 (0.1)	0 (0)	14 (0.1)
BMI, kg/m^2^	23.3 (4.0)	22.9 (3.7)	22.3 (3.7)	23.0 (3.9)	23.3 (4.4)	23.5 (4.3)	23.1 (4.3)	23.3 (4.4)
Waist circumference, cm	84 (11)	85 (11)	84 (11)	84 (11)	74 (10)	77 (10)	77 (10)	75 (10)
Body fat percentage	20 (7)	21 (7)	21 (7)	20 (7)	32 (8)	34 (8)	34 (8)	33 (8)
SBP, mmHg	125 (14)	131 (18)	136 (20)	129 (17)	122 (15)	133 (19)	139 (21)	127 (19)
FEV1/FVC <70%	3482 (34)	1448 (35)	1343 (36)	6273 (35)	3639 (29)	1289 (27)	882 (26)	5810 (28)
Mean (of right and left) grip strength, kg	34 (8)	28 (7)	24 (6)	30 (8)	21 (5)	18 (5)	16 (4)	20 (5)
Prevalence of one or more chronic diseases^a^	1012 (10)	695 (17)	768 (21)	2475 (14)	1113 (9)	875 (19)	877 (26)	2865 (14)
Poor self-reported health	32 (0.3)	28 (1)	32 (1)	92 (1)	59 (0.5)	58 (1)	47 (1)	164 (1)
Depressive symptoms^b^	5730 (56)	2218 (54)	1970 (53)	9918 (55)	7505 (60)	2897 (62)	1965 (59)	12 367 (60)
Feeling more sad/depressed than usual	1986 (20)	835 (20)	754 (20)	3575 (20)	3427 (28)	1449 (31)	980 (29)	5856 (29)
Loss of interest in hobbies/activities	2451 (24)	918 (22)	876 (24)	4245 (24)	3535 (28)	1319 (28)	973 (29)	5827 (28)
Loss of appetite for favourite food	3106 (31)	1226 (30)	1045 (28)	5377 (30)	4033 (32)	1621 (35)	1083 (33)	6737 (33)
Feeling worthless and useless	3393 (33)	1271 (31)	1090 (29)	5754 (32)	3926 (32)	1450 (31)	927 (28)	6303 (31)

Data are *n* (%) or mean (SD).

BMI, body mass index; SBP, systolic blood pressure; FEV1, forced expiratory volume in one s; FVC, forced vital capacity.

a Based on self-reported personal medical history (any of hypertension, diabetes, heart disease, stroke, asthma, chronic bronchitis, liver disease, kidney disease, cancer, gallstone/gallbladder, peptic ulcer, tuberculosis).

b At least one symptom of depression: feeling much more sad or depressed than usual (25%), loss of interest in most things like hobbies or activities that usually give pleasure (26%), feeling so hopeless that lost appetite for favourite food (32%), or feeling worthless or useless (31%) for a period of 2 or more weeks in the past 12 months.

**Figure 2.**
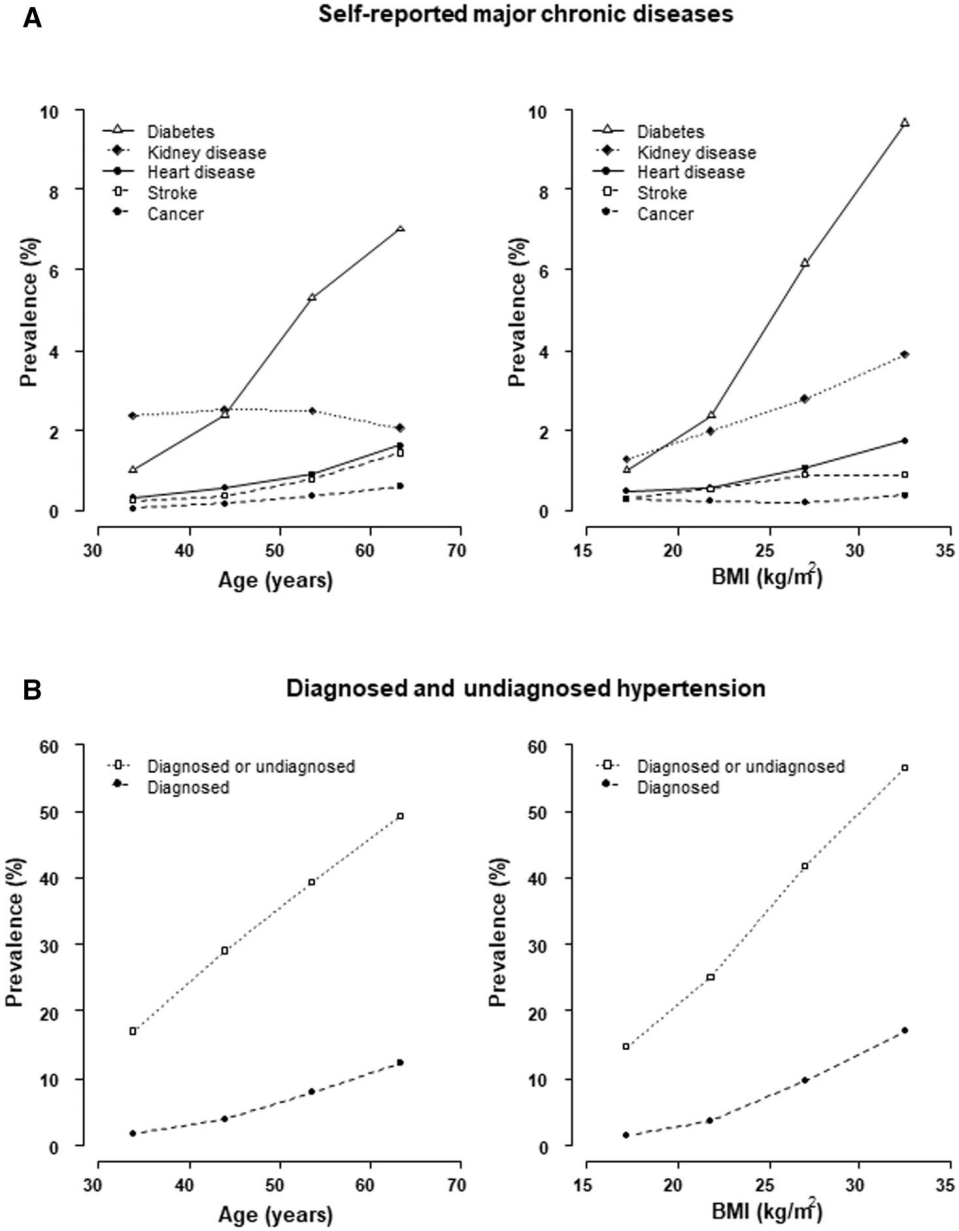
Prevalence of (A) self-reported major chronic diseases and (B) diagnosed and undiagnosed hypertension among 38 442 study participants, by age and body mass index (BMI), standardized for sex and, where appropriate, age. Undiagnosed hypertension: systolic blood pressure ≥140 mmHg or diastolic blood pressure ≥90 mmHg

Previously diagnosed chronic disease was reported by 14% of participants (Table 3). Hypertension was most commonly reported (6%), with women twice as likely as men to have been diagnosed across all ages, followed by diabetes (4% of men and 3% of women) (Supplementary Table S4, available as Supplementary data at *IJE* online). A prior diagnosis of chronic kidney disease was reported by 3% of men and 2% of women, and 1% of participants reported pre-existing chronic heart disease or stroke. A diagnosis of cancer was uncommon (<1%). In addition to the 6% of participants with self-reported hypertension, a further 26% had undiagnosed hypertension. With the exception of chronic kidney disease, for which there was little variation in prevalence across age groups, the prevalence of these chronic diseases increased with increasing age, with particularly strong associations of age with diabetes and hypertension (Figure 2). Previous analyses among a subset of the study population have shown a strong positive association of adiposity with both blood pressure and prevalent hypertension,^9^ replicated in this larger population. BMI was also strongly positively associated with diabetes prevalence and, to a lesser extent, with prevalent chronic kidney and cardiovascular diseases.

A striking characteristic of this cohort is the high frequency of depressive symptoms (Table 3). More than half of participants (55% of men and 60% of women) reported one or more depressive symptom, varying little with age. Feeling worthless or useless and loss of appetite for a period of at least 2 weeks in the past year were reported by approximately one-third of men and women. Feeling more sad or depressed than usual, and loss of interest in hobbies and activities that usually give pleasure, were reported by one-quarter of participants, more commonly among women (29% and 28%, respectively) than men (20% and 24%, respectively). Further research will be essential to understand these high prevalences, including exploration of the population-specific relevance and validity of depression assessment tools.

## What are the main strengths and weaknesses?

ISHA is the only blood-based prospective cohort study in India. Moreover, once baseline data collection is completed it will be the largest prospective cohort study in South Asia. Although not nationally-representative, this would not be expected to bias estimated associations, which would be expected to be generalizable given the large and diverse study population.^10^^,^^11^ Furthermore, the focus on a predominantly rural and currently understudied population with unique lifestyle and environmental exposures will provide real opportunity to advance understanding of the determinants of disease. For example, the relatively high ischaemic heart disease mortality rates in this relatively lean population, with a comparably low prevalence of smoking, and opportunity for assessment of blood-based risk factors provide scope for addressing persisting uncertainties. These include those relating to mechanisms underlying the observed absence of association of BMI with cardiac mortality in the Chennai Prospective Study of 0.5 million adults, despite a strong positive association of BMI with blood pressure,^12^ and the rising rates of cardiac mortality in rural India.^1^ The data collected seek to maximize such insights gained, given the specific characteristics of the study population. Enrolment at household level would be expected to reduce potential healthy volunteer bias, and the use of laptops with direct data entry ensures reliable and efficient collection of these data. The repeat baseline assessments undertaken among a subset of participants within months of the original assessment provide a valuable opportunity for data collection quality control. These have shown a high level of agreement between the two assessments (>90% for self-reported prior chronic disease diagnoses and ∼85% for major lifestyle factors). The COVID-19 pandemic imposed significant delays on recruitment and data collection, and may have precluded inclusion of some enumerated participants in subsequent data collection. However, the multiple sources of follow-up, capturing both fatal and non-fatal disease events, will provide an invaluable opportunity to study the determinants and consequences of COVID-19 and other diseases. Given the relatively young average age of participants, it may take time to accrue deaths and non-fatal disease events. However, this also ensures a high proportion of disease-free participants at recruitment, affording the greatest opportunity for investigation of risk factors for chronic diseases. Finally, collection of blood samples for long-term storage will facilitate assessment of the role of genetic and other blood-based factors in determining disease risks and prognosis, providing opportunity for enhanced prevention, prediction and treatment of diverse diseases in the population of India and more widely.

Planned expansion of ISHA to additional sites in diverse areas of India, including Varanasi (Uttar Pradesh), Guwahati (Assam), Sangrur (Punjab) and Mullanpur (Punjab) (Supplementary Figure S10, available as Supplementary data at *IJE* online), will further strengthen the study.

## Can I get hold of the data? Where can I find out more?

Detailed data collection from the full study population of 219 888 is expected to be completed by December 2026. Although the study data are not yet freely available, once data collection is complete, specific proposals for future collaboration are welcome, addressed to the study’s International Steering Committee (e-mail: isha@tmc.gov.in).

## Ethics approval

The study gained approval from the Research Ethics Committee of the Tata Memorial Centre (IEC/560) and Nargis Dutt Memorial Cancer Hospital, Barshi (RCR/33/2008).

## Supplementary Material

dyae079_Supplementary_Data

## Data Availability

See ‘Can I get hold of the data? Where can I find out more?’ above.
