# Supplementary material for: Cohort Profile: Indian Study of Healthy Ageing (ISHA-Barshi)
Source: Int J Epidemiol. 2024 Jun 14;53(4):dyae079. doi: 10.1093/ije/dyae079 (PMC11180225; doi:10.1093/ije/dyae079)
Supplement: dyae079_Supplementary_Data [file dyae079_supplementary_data.pdf]

Cohort Profile: Indian Study of Healthy Ageing (ISHA)

Supplementary Material, Table of Contents

	Page
Supplementary Figure S1: Map showing locations of Solapur District and Barshi	2
Supplementary Figure S2: Indian Study of Healthy Ageing structure	3
Supplementary Figure S3: Indian Study of Healthy Ageing baseline survey and resurvey	4
Supplementary Figure S4: Indian Study of Healthy Ageing mortality data collection	5
Supplementary Figure S5: Indian Study of Healthy Ageing record linkage	6
Supplementary Figure S6: Indian Study of Healthy Ageing active follow-up	7
Supplementary Figure S7: Indian Study of Healthy Ageing Health Check-Up Camp	8
Supplementary Figure S8: Indian Study of Healthy Ageing data capture and syncing overview	9
Supplementary Figure S9: Indian Study of Healthy Ageing data capture and syncing	10
Supplementary Figure S10: Map showing locations of additional Indian Study of Healthy Ageing study locations	11
Supplementary Table S1: Characteristics of 119 387 study households	12
Supplementary Table S2: Age and sex of 219 888 recruited participants and 17 362 eligible non-participants from the same households	13
Supplementary Table S3: Baseline distributions of selected disease risk factors in 38 442 participants, by age and sex	14
Supplementary Table S4: Baseline characteristics of 38 442 study participants, by age and sex	15

Supplementary Figure S1: Map showing locations of Solapur District and Barshi

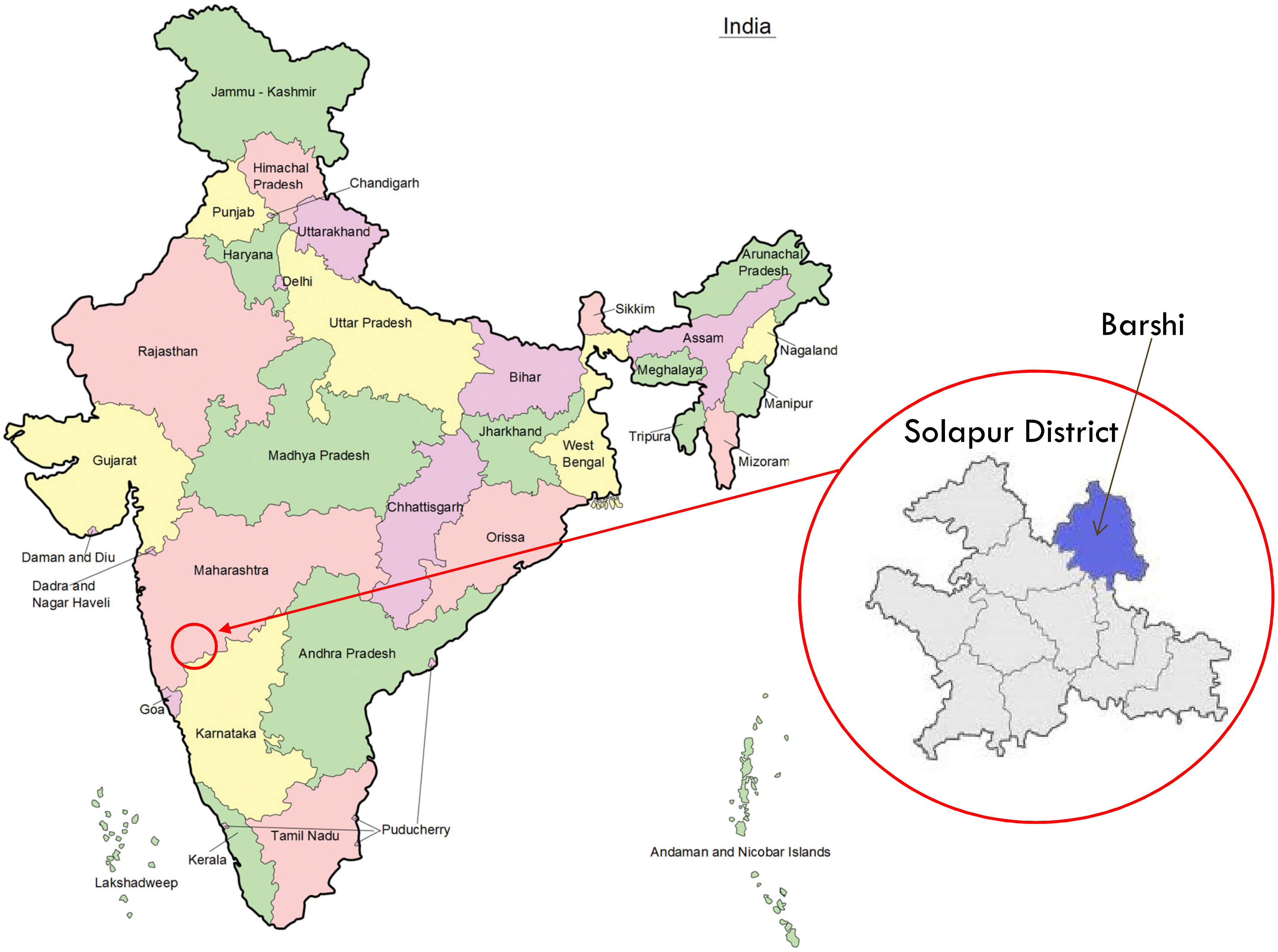

Supplementary Figure S2: Indian Study of Healthy Ageing structure

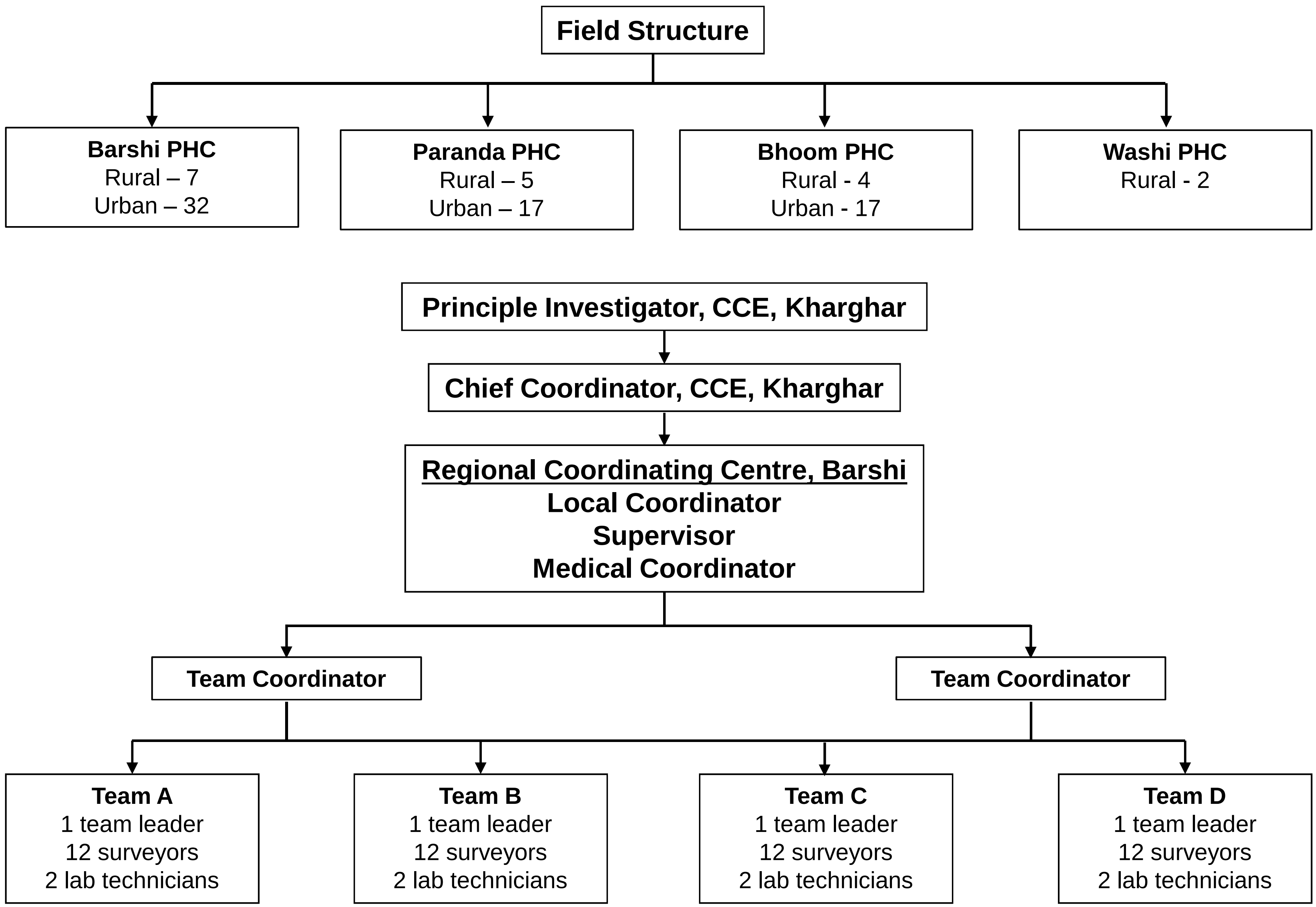

CCE = Centre for Cancer Epidemiology; PHC = primary healthcare centres

Supplementary Figure S3: Indian Study of Healthy Ageing baseline survey and resurvey

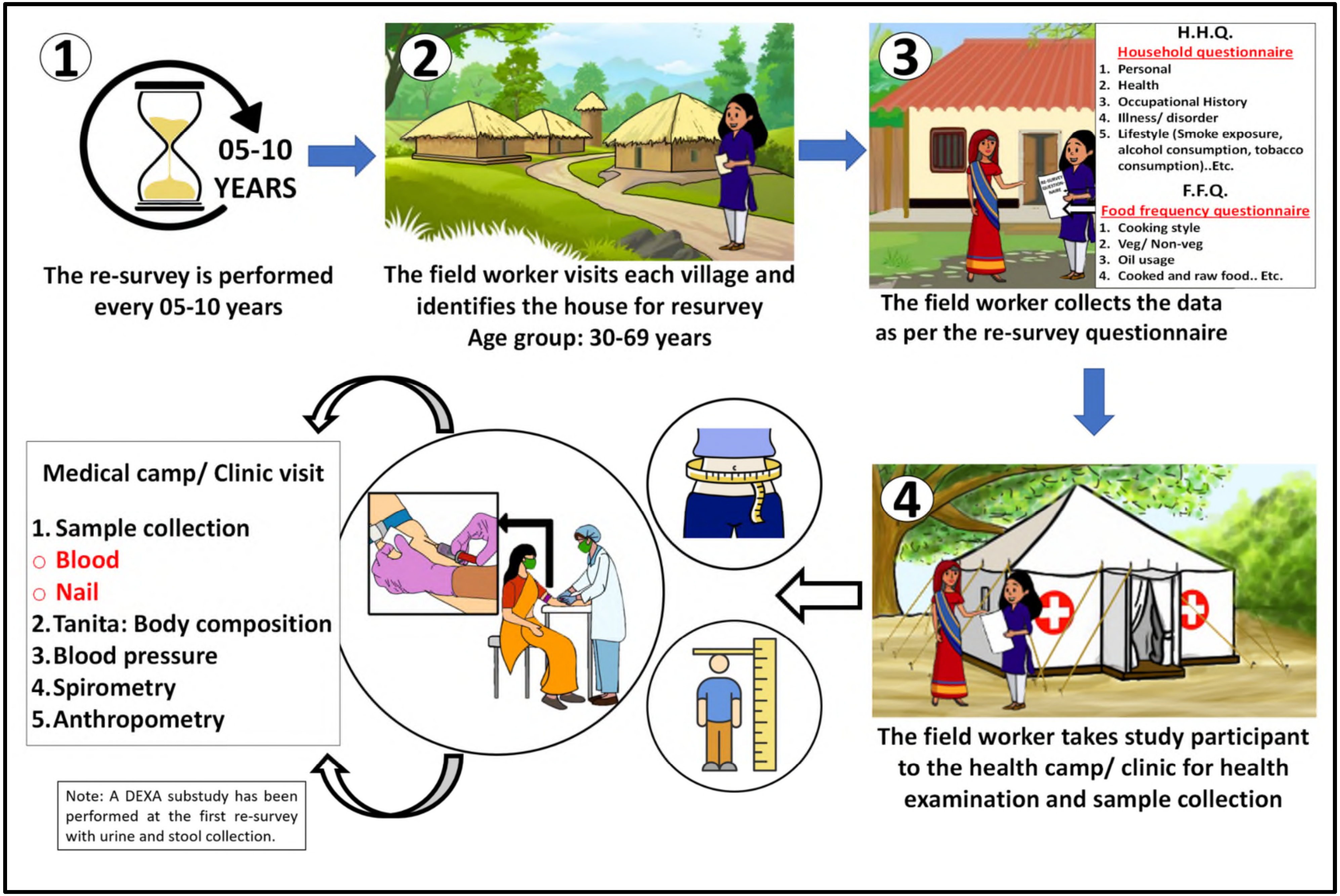

DEXA = dual-energy absorptiometry; FFQ = food frequency questionnaire; HHQ = household questionnaire

Supplementary Figure S4: Indian Study of Healthy Ageing mortality data collection

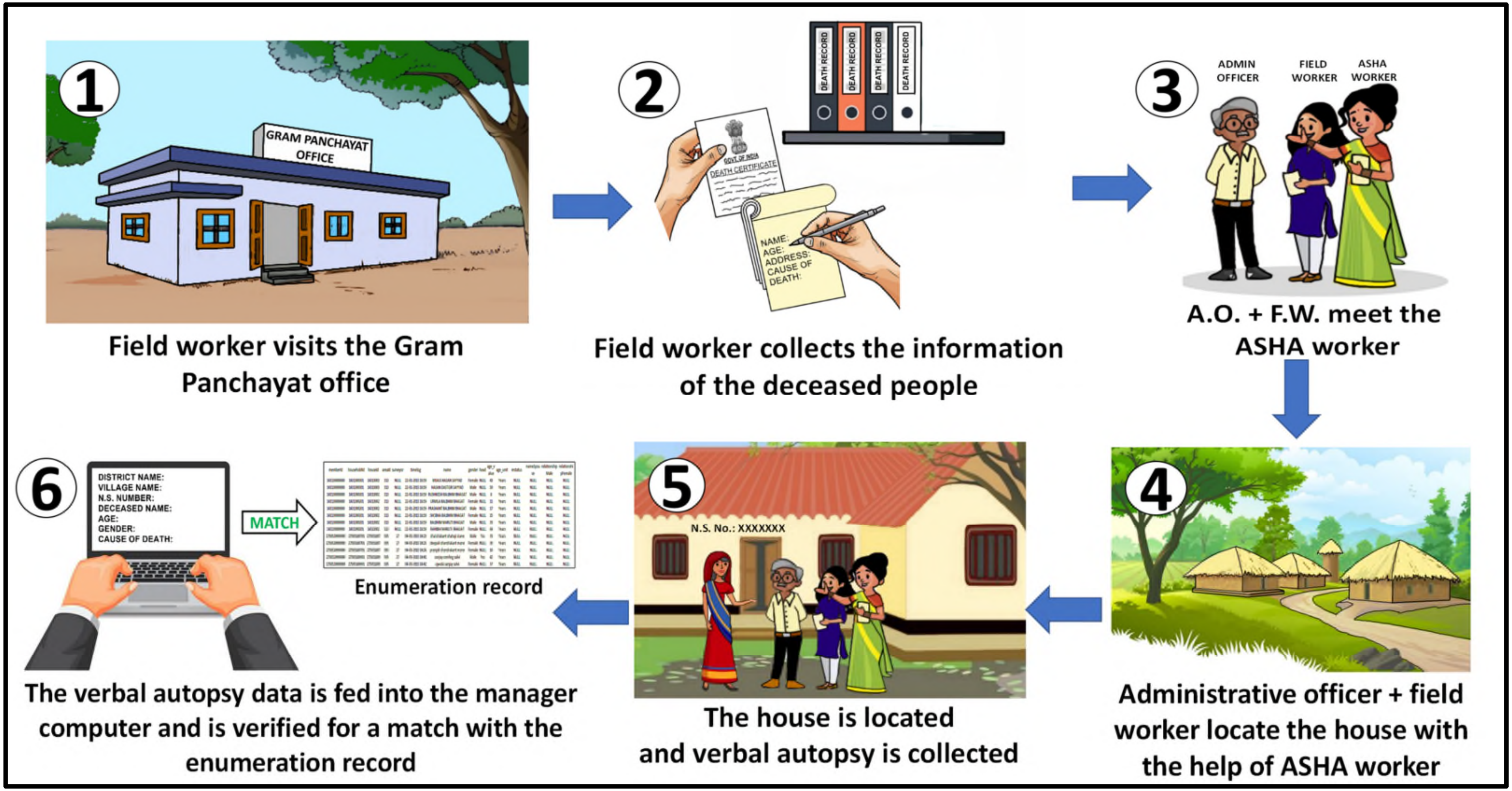

AO = admin officer; ASHA = Accredited Social Health Activist; FW = field worker

Supplementary Figure S5: Indian Study of Healthy Ageing record linkage

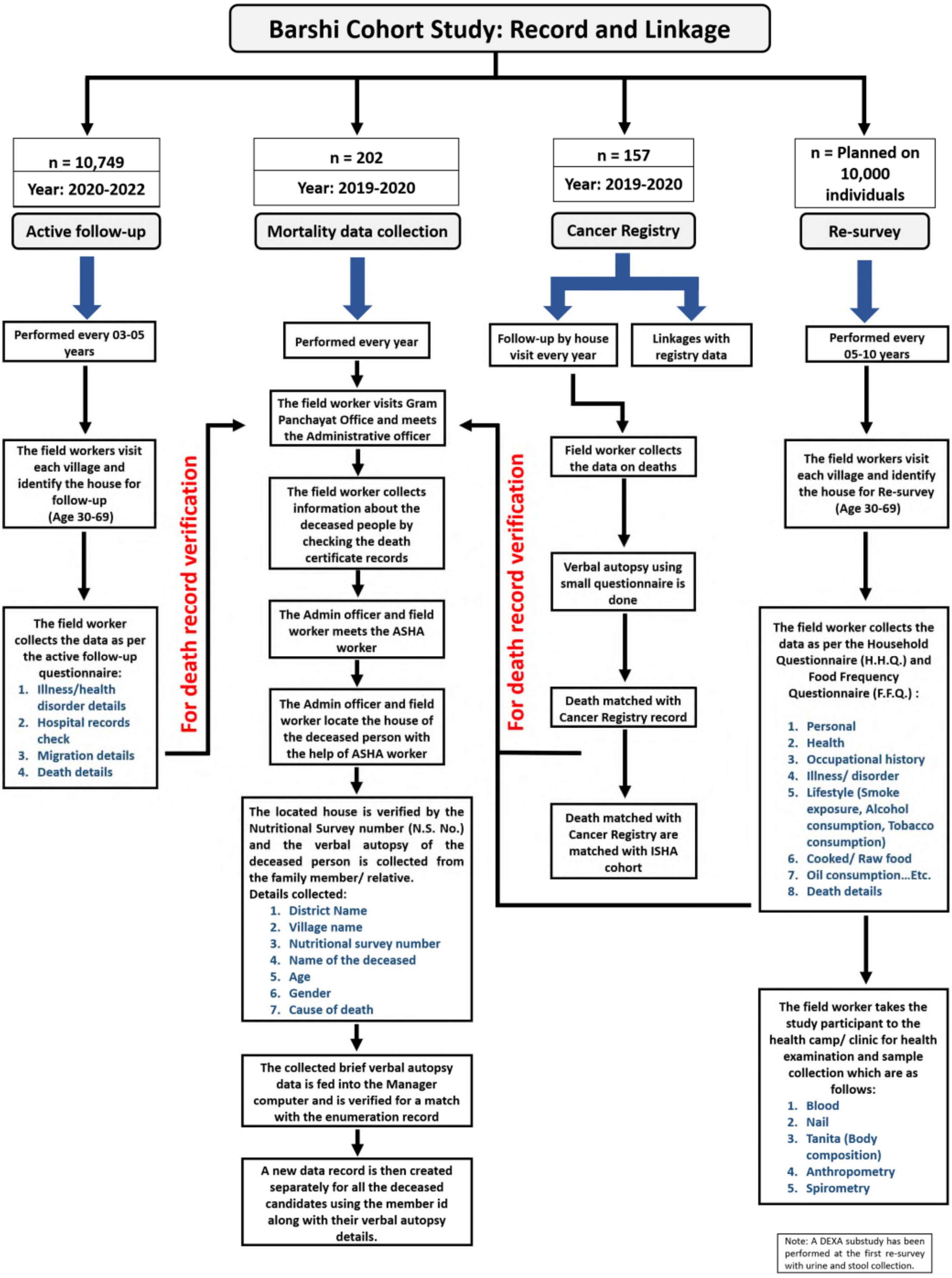

DEXA = dual-energy absorptiometry

Supplementary Figure S6: Indian Study of Healthy Ageing active follow-up

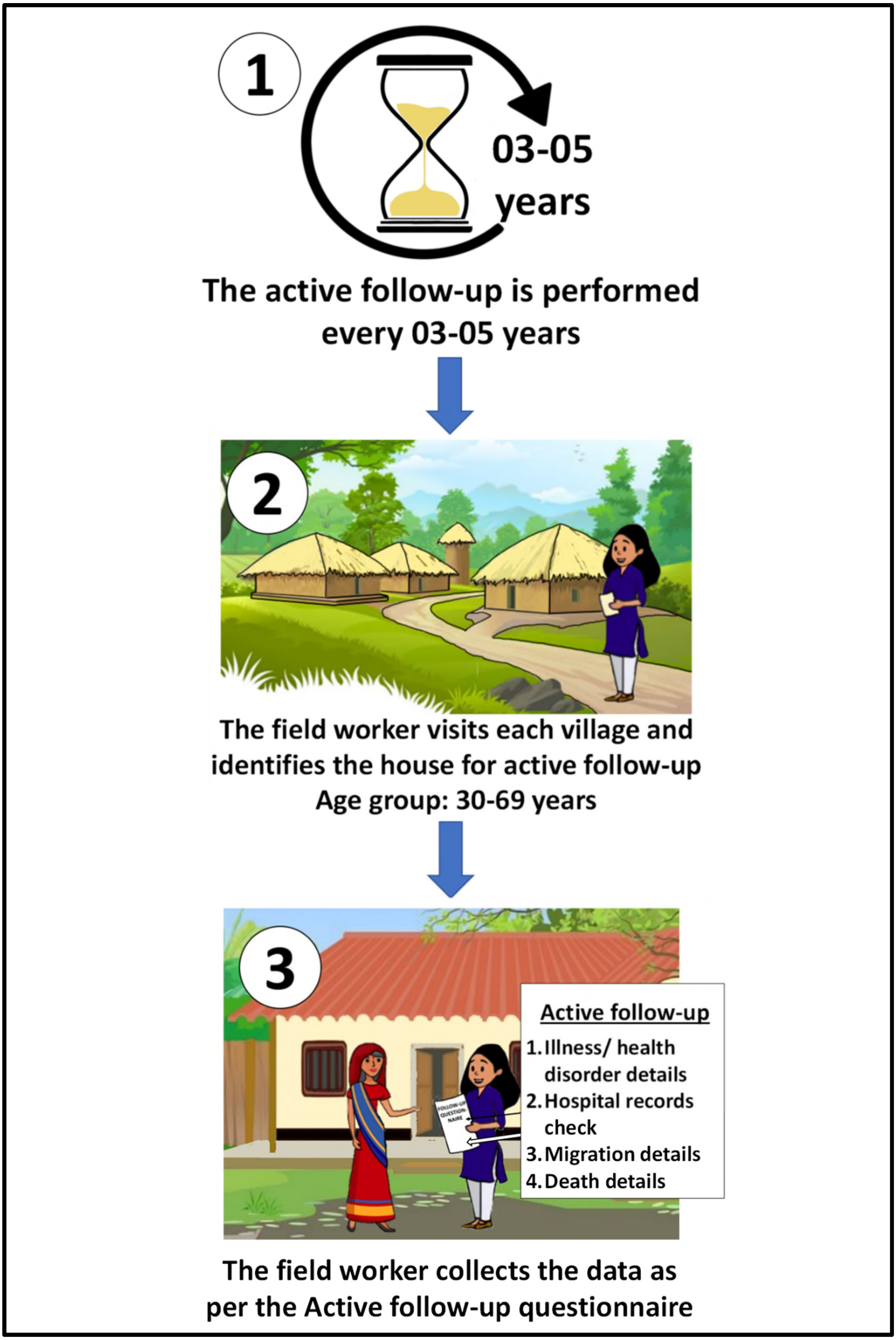

Supplementary Figure S7: Indian Study of Healthy Ageing Health Check-Up Camp

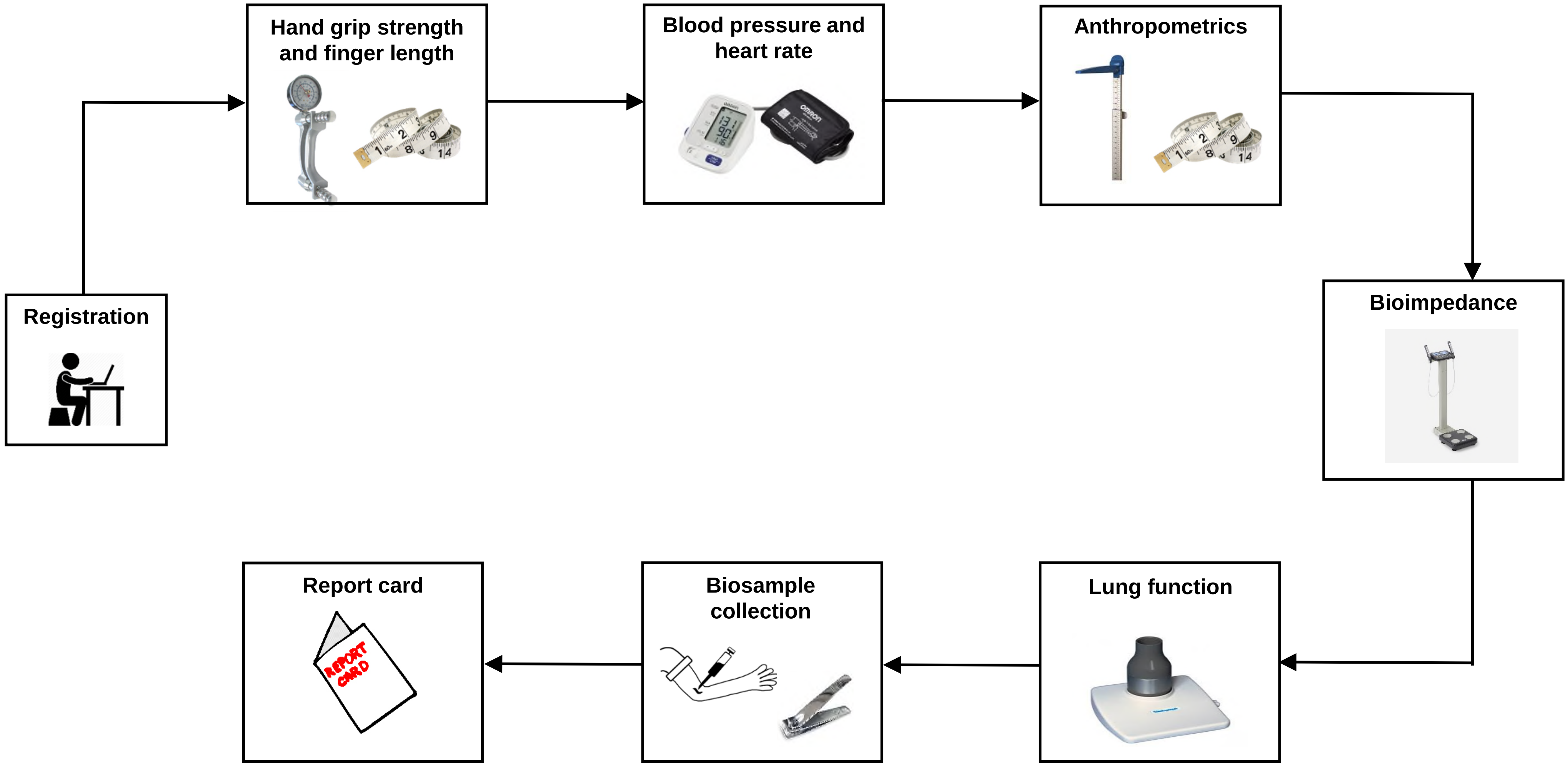

Supplementary Figure S8: Indian Study of Healthy Ageing data capture and syncing overview

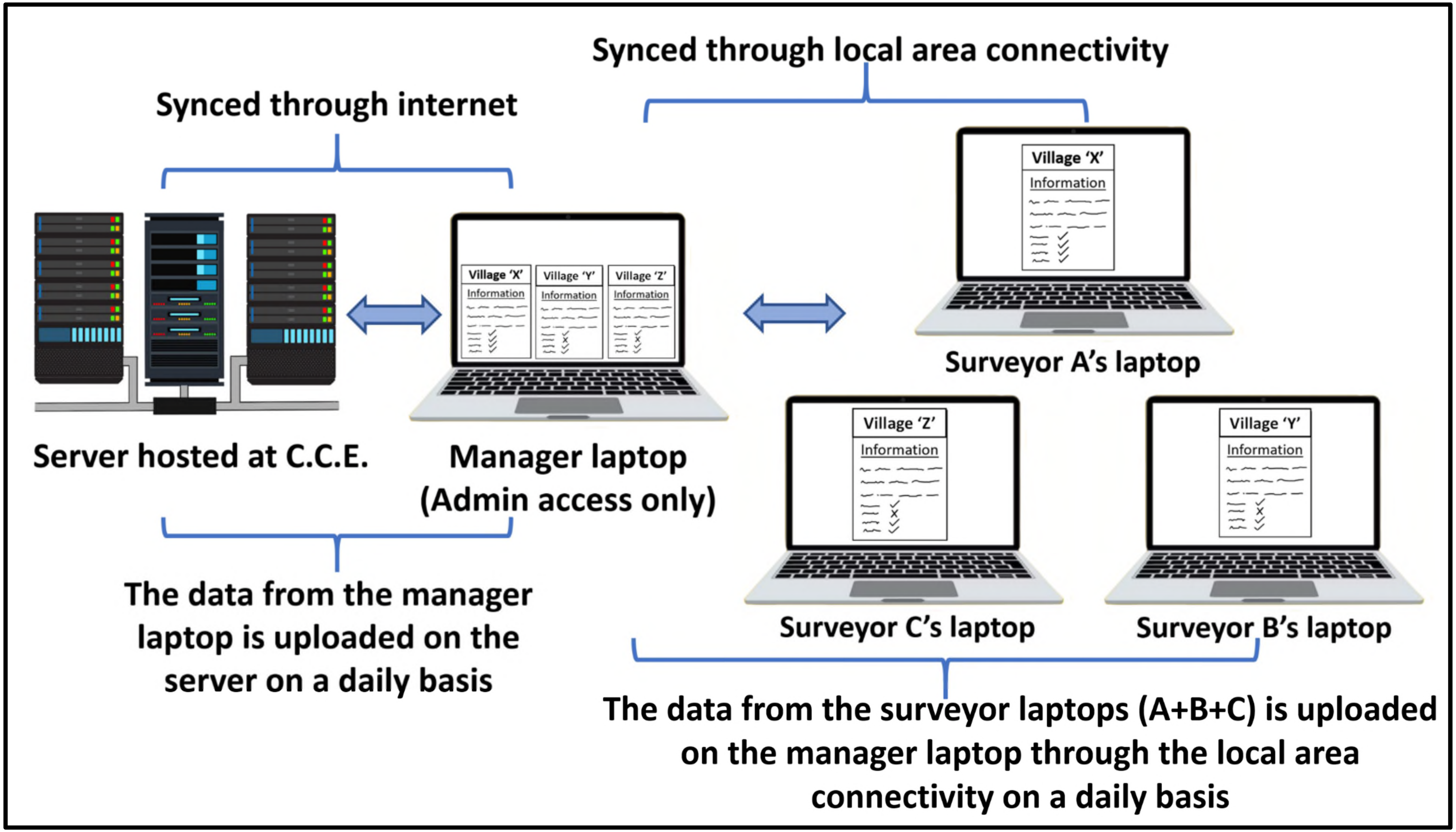

CCE = Centre for Cancer Epidemiology

Supplementary Figure S9: Indian Study of Healthy Ageing data capture and syncing

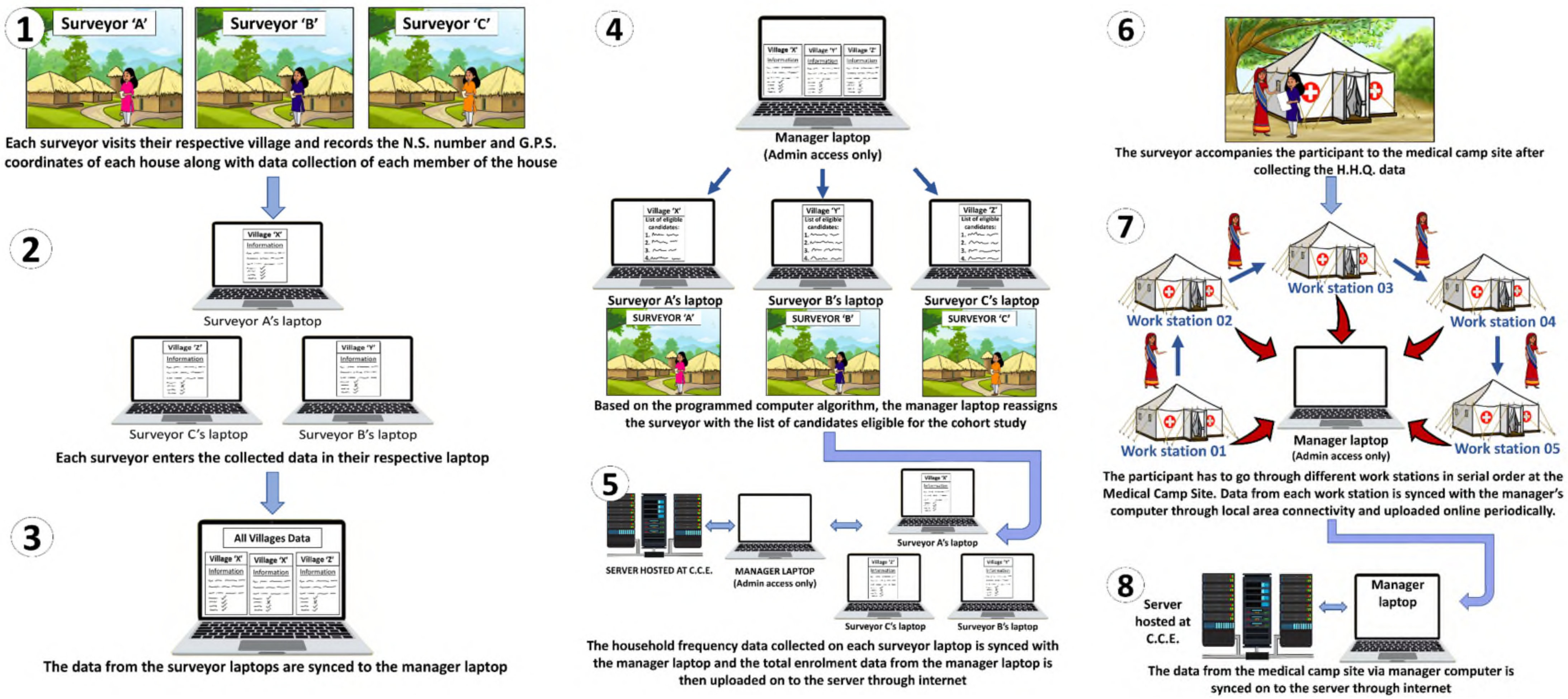

CCE = Centre for Cancer Epidemiology; GPS = Global Positioning System; HHQ = household questionnaire

Supplementary Figure S10: Map showing locations of additional Indian Study of Healthy Ageing study locations

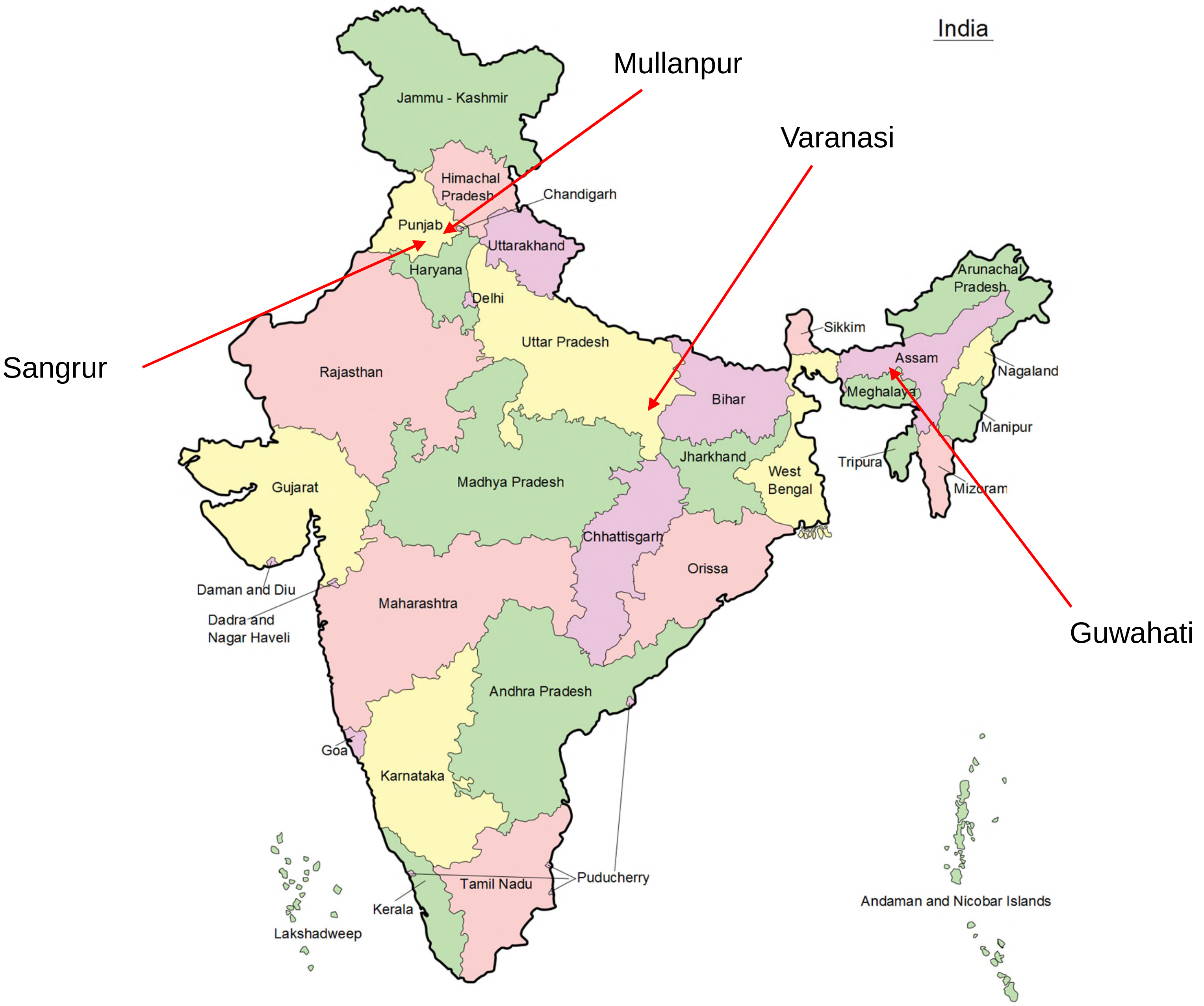

Supplementary Table S1: Characteristics of 119 387 study households

	Households n (%) or mean (SD)
Household size[a]	
1	5680 (5)
2	14 103 (12)
3	12 888 (11)
4	25 390 (21)
5	19 067 (16)
6	13 831 (12)
7	6557 (5)
8+	9047 (8)
Religion	
Hindu	96 936 (81)
Muslim	8639 (7)
Jain	564 (0.5)
Other/not known	13 248 (11)
Type of house	
Pucca	29 891 (25)
Kuccha	23 693 (20)
Mixed	60 588 (51)
Not known	5215 (4)
Drinking water source	
Well	14 449 (12)
Hand pump	19 671 (16)
Shared tap	15 666 (13)
Own tap	44 708 (37)
Other/not known	24 893 (21)
Exposure to domestic air pollution (%)	
Use of smoky cooking fuel[b]	45 623 (38)
Home smoky due to cooking[bc]	19 564 (16)
Household cooking, monthly consumption	
Onion, kg	3.3 (2.1)
Garlic, bulbs	36.9 (20.3)
Turmeric, g	67.4 (59.8)
Gingelly oil, l	0.04 (0.61)
Groundnut oil, l	0.1 (0.7)
Mustard oil, l	0.05 (0.6)
Palm oil, l	2.4 (2.7)
Sunflower oil, l	1.6 (2.7)
Other oil, l	0.4 (1.6)

[a]For 12 824 households size was unknown.

[b]Restricted to households where cooking is undertaken indoors.

[c]One or more of charcoal, wood, cowdung cake, coal, kerosene or agricultural waste.

Supplementary Table S2: Age and sex of 219 888 recruited participants and 17 362 eligible non-participants from the same households

	Men, by age (years)				Women, by age (years)			
	30-49	50-59	60-69	All	30-49	50-59	60-69	All
**Recruited participants**								
Number of participants	62 722 (57)	24 706 (23)	21 656 (20)	109 084 (100)	63 693 (57)	24 754 (22)	22 357 (20)	110 804 (100)
Age, years (mean (SD))	38 (6)	54 (3)	63 (3)	47 (11)	39 (6)	54 (3)	63 (3)	47 (11)
**Non-participants**								
Number of non-participants	5280 (56)	2012 (21)	2220 (23)	9512 (100)	3852 (49)	1674 (21)	2324 (30)	7850 (100)
Age, years (mean (SD))	38 (6)	54 (3)	64 (3)	47 (12)	39 (6)	54 (3)	64 (3)	49 (12)

Data are n (%) or mean (SD).

Supplementary Table S3: Baseline distributions of selected disease risk factors in 38 442 participants, by age and sex

	Men, by age (years)				Women, by age (years)			
	30-49	50-59	60-69	All	30-49	50-59	60-69	All
Number of participants	10 170 (57)	4083 (23)	3710 (21)	17 963 (100)	12 454 (61)	4694 (23)	3331 (16)	20 479 (100)
Current only chew	5541 (54)	2787 (68)	2745 (74)	11 073 (62)	938 (8)	867 (18)	766 (23)	2571 (13)
Current only smoke	65 (1)	68 (2)	50 (1)	183 (1)	1 (0.01)	1 (0.02)	3 (0.1)	5 (0.02)
Current chew and smoke	196 (2)	135 (3)	121 (3)	452 (3)	0 (0)	0 (0)	1 (0.03)	1 (0.01)
Current chewers	5733 (56)	2923 (72)	2865 (77)	11 521 (64)	936 (8)	867 (18)	767 (23)	2570 (13)
Chewing tobacco	1420 (14)	657 (16)	703 (19)	2780 (15)	50 (0.4)	82 (2)	87 (3)	219 (1)
Khaini or tobacco, lime mix	3982 (39)	2091 (51)	1887 (51)	7960 (44)	149 (1)	203 (4)	220 (7)	572 (3)
Ghutka or tobacco lime, areca nut mix	1951 (19)	322 (8)	137 (4)	2410 (13)	18 (0.1)	7 (0.1)	7 (0.2)	32 (0.2)
Oral tobacco	34 (0.3)	39 (1)	34 (1)	107 (1)	661 (5)	548 (12)	422 (13)	1631 (8)
Nasal snuff/misri	3 (0.03)	7 (0.2)	2 (0.1)	12 (0.1)	23 (0.2)	21 (0.4)	26 (1)	70 (0.3)
Other smokeless tobacco product	356 (4)	401 (10)	470 (13)	1227 (7)	32 (0.3)	27 (1)	50 (2)	109 (1)
Pan-masala and betel quid without tobacco	693 (7)	670 (16)	857 (23)	2220 (12)	96 (1)	86 (2)	85 (3)	267 (1)
Ex- chewers	189 (2)	156 (4)	176 (5)	521 (3)	129 (1)	106 (2)	108 (3)	343 (2)
Never chewers	4221 (42)	990 (24)	655 (18)	5866 (33)	11 350 (91)	3708 (79)	2447 (73)	17 505 (85)
Current smokers	261 (3)	203 (5)	171 (5)	635 (4)	1 (0.01)	1 (0.02)	4 (0.1)	6 (0.03)
Cigarette	213 (2)	153 (4)	101 (3)	467 (3)	0 (0)	0 (0)	0 (0)	0 (0)
Bidi	42 (0.4)	34 (1)	47 (1)	123 (1)	0 (0)	1 (0.02)	4 (0.1)	5 (0.02)
Other	28 (0.3)	28 (1)	35 (1)	91 (1)	1 (0.01)	0 (0)	0 (0)	1 (0.01)
Ex- smokers	256 (3)	239 (6)	260 (7)	755 (4)	2 (0.02)	6 (0.1)	1 (0.03)	9 (0.04)
Never smokers	9615 (95)	3622 (89)	3262 (88)	16 499 (92)	12 405 (100)	4668 (99)	3310 (99)	20 383 (100)
Current alcohol drinkers	1488 (15)	694 (17)	625 (17)	2807 (16)	5 (0.04)	1 (0.02)	0 (0)	6 (0.03)
Only country liquor/arrack/sugar cane	664 (7)	498 (12)	474 (13)	1636 (9)	4 (0.03)	0 (0)	0 (0)	4 (0.02)
Only spirit (rum, whisky, gin, vodka)	492 (5)	130 (3)	91 (2)	713 (4)	0 (0)	1 (0.02)	0 (0)	1 (0.01)
Other	332 (3)	66 (2)	60 (2)	458 (3)	1 (0.01)	0 (0)	0 (0)	1 (0.01)
Ex- drinkers	780 (8)	473 (12)	441 (12)	1694 (9)	6 (0.05)	2 (0.04)	0 (0)	8 (0.04)
Never drank	7852 (77)	2895 (71)	2628 (71)	13 375 (74)	12 404 (100)	4679 (100)	3319 (100)	20 402 (100)
BMI								
<18.5 (%)	799 (8)	346 (8)	441 (12)	1586 (9)	1285 (10)	437 (9)	361 (11)	2083 (10)
18.5-<25 (%)	4322 (42)	1950 (48)	1918 (52)	8190 (46)	5499 (44)	2060 (44)	1520 (46)	9079 (44)
25-<30 (%)	2045 (20)	778 (19)	549 (15)	3372 (19)	2579 (21)	1046 (22)	636 (19)	4261 (21)
>=30 (%)	412 (4)	114 (3)	94 (3)	620 (3)	729 (6)	275 (6)	177 (5)	1181 (6)
Standing height, cm	164 (6)	162 (6)	161 (6)	163 (6)	151 (6)	149 (6)	148 (6)	150 (6)
Sitting height, cm	124 (4)	122 (4)	121 (4)	123 (4)	117 (4)	115 (4)	114 (4)	116 (4)
Hip circumference, cm	92 (8)	90 (7)	90 (7)	91 (8)	93 (9)	93 (9)	92 (9)	93 (9)
Waist-to-hip ratio	0.92 (0.07)	0.94 (0.07)	0.93 (0.08)	0.92 (0.07)	0.80 (0.06)	0.82 (0.06)	0.83 (0.06)	0.81 (0.06)
DBP, mmHg	82 (10)	84 (11)	84 (11)	83 (10)	82 (10)	86 (11)	86 (11)	84 (10)

Data are n (%) or mean (SD).

BMI=body mass index; DBP=diastolic blood pressure.

Chewing and smoking proportions will sum to >100% since some participants chew/smoke more than one type of product.

Supplementary Table S4: Baseline characteristics of 38 442 study participants, by age and sex

	Men, by age (years)				Women, by age (years)			
	**30-49**	**50-59**	**60-69**	**All**	**30-49**	**50-59**	**60-69**	**All**
Number of participants	10 170 (57)	4083 (23)	3710 (21)	17 963 (100)	12 454 (61)	4694 (23)	3331 (16)	20 479 (100)
Age, years (mean (SD))	38 (6)	54 (3)	63 (3)	47 (11)	38 (6)	53 (3)	63 (3)	46 (11)
Prevalence of chronic diseases								
Hypertension	198 (2)	220 (5)	310 (8)	728 (4)	406 (3)	471 (10)	527 (16)	1404 (7)
Diabetes	184 (2)	219 (5)	237 (6)	640 (4)	182 (1)	248 (5)	252 (8)	682 (3)
Heart disease	41 (0.4)	41 (1)	66 (2)	148 (1)	59 (0.5)	39 (1)	49 (1)	147 (1)
Stroke	32 (0.3)	42 (1)	72 (2)	146 (1)	37 (0.3)	27 (1)	34 (1)	98 (0.5)
Asthma	59 (1)	46 (1)	83 (2)	188 (1)	100 (1)	101 (2)	103 (3)	304 (1)
Chronic bronchitis	23 (0.2)	16 (0.4)	21 (1)	60 (0.3)	24 (0.2)	16 (0.3)	9 (0.3)	49 (0.2)
Chronic liver disease	123 (1)	52 (1)	39 (1)	214 (1)	101 (1)	29 (1)	24 (1)	154 (1)
Chronic kidney disease	337 (3)	133 (3)	97 (3)	567 (3)	206 (2)	85 (2)	52 (2)	343 (2)
Cancer	4 (0.04)	10 (0.2)	13 (0.4)	27 (0.2)	21 (0.2)	23 (0.5)	28 (1)	72 (0.4)
Gallstone/gallbladder disorder	36 (0.4)	16 (0.4)	12 (0.3)	64 (0.4)	35 (0.3)	17 (0.4)	15 (0.5)	67 (0.3)
Peptic ulcer disease	24 (0.2)	19 (0.5)	17 (0.5)	60 (0.3)	21 (0.2)	7 (0.1)	10 (0.3)	38 (0.2)
Tuberculosis	68 (1)	42 (1)	50 (1)	160 (1)	64 (1)	30 (1)	21 (1)	115 (1)

Data are n (%) or mean (SD).
